# Mechanisms of Tactile Sensory Phenotypes in Autism: Current Understanding and Future Directions for Research

**DOI:** 10.1007/s11920-019-1122-0

**Published:** 2019-12-05

**Authors:** Melanie D. Schaffler, Leah J. Middleton, Ishmail Abdus-Saboor

**Affiliations:** Department of Biology, University of Pennsylvania, Philadelphia, PA 19104 USA

**Keywords:** Autism, Somatosensation, Tactile deficits, Peripheral nervous system, Touch, Pain

## Abstract

**Purpose of Review:**

This review aims to summarize the current body of behavioral, physiological, and molecular knowledge concerning tactile sensitivity in autism spectrum disorder (ASD), with a focus on recent studies utilizing rodent models.

**Recent Findings:**

Mice with mutations in the ASD-related genes, Shank3, Fmr1, UBE3A, and Mecp2, display tactile abnormalities. Some of these abnormalities appear to be caused by mutation-related changes in the PNS, as opposed to changes in the processing of touch stimuli in the CNS, as previously thought. There is also growing evidence suggesting that peripheral mechanisms may contribute to some of the core symptoms and common comorbidities of ASD. Researchers are therefore beginning to assess the therapeutic potential of targeting the PNS in treating some of the core symptoms of ASD.

**Summary:**

Sensory abnormalities are common in rodent models of ASD. There is growing evidence that sensory hypersensitivity, especially tactile sensitivity, may contribute to social deficits and other autism-related behaviors.

## Introduction

Autism spectrum disorder (ASD) is a prevalent neurodevelopmental disorder characterized by social deficits and stereotyped repetitive behaviors [1]. In addition to these core features of autism, researchers have reported that approximately 95% of individuals with ASD exhibit sensory abnormalities, with ~ 60% displaying altered tactile sensitivity [2]. Some individuals injure themselves by head banging, skin picking, and self-biting [3, 4], though the role of pain sensitivity in self-injury is unclear [3, 5–7]. Others display exaggerated responses to touch and pain [8–12]. Some individuals will simultaneously display hypersensitivity to some stimuli and hyposensitivity to others, often depending on the context [13–16]. For example, individuals may report hating hugs, while seeking out deep tactile pressure in other ways [17]. While it is possible that the social component of hugs is what some individuals find repulsive, the reasons why individuals may display both hypo- and hypersensitivity in a variety of situations are not well understood.

Compared with research on the core symptoms of autism, little work has been done investigating the mechanisms behind the tactile abnormalities associated with ASD. However, these tactile abnormalities may very well contribute to some of the core symptoms. For example, hypersensitivity of peripheral neurons may contribute to avoidance of social touch, a common behavioral phenotype in individuals with ASD [18, 19]. Hyposensitivity in the peripheral nervous system may result in an inadequate amount of touch information reaching the brain, causing individuals to be indifferent to social touch. Understanding the causes and effects of somatosensory differences is therefore imperative to understanding the etiology of the disorder and its symptoms. We will review the recent literature on tactile and pain sensitivity in ASD, focusing on studies using genetic rodent models of ASD, as rodent models allow us to directly assess the physiological and behavioral effects of specific autism-related genetic mutations in a controlled environment. Additionally, having the complete genetic and single nucleotide polymorphism (SNP) maps in preclinical rodent models permits a faster translation of research findings in terms of linking a genetic variant with a specific ASD-like phenotype. Lastly, as emerging technologies such as CRISPR/Cas9 permit precise modification of virtually any genome including rodents, researchers will increasingly have the ability to model human ASD-related disease alleles in mouse models while testing for ASD phenotypes in behaving mice [20].

## The Contribution of Sensory Deficits to Autism

The sense of touch is essential for sensorimotor control and exploration of our environment. Touch is also a major component of social interactions and the formation of relationships. Social touch, especially during development, can have long-term positive effects across species. Touch communication between mothers and infants improves affect and reduces cortisol reactivity [21, 22]. Premature infants isolated in incubators who receive 45 min of touch daily score higher on cognitive and motor tests at 6 months [23, 24]. Mothers who not only touch their infants but also rock them induce a calming response in the infant and interestingly; this calming behavior induced by maternal carrying and moving of offspring is recapitulated in mice [25]. The mom’s induction of the calming response in neonatal mice by retrieving and walking with the pups is dependent upon tactile stimulation, with an additional input from vestibular-proprioceptive input [25]. Additionally, rats that received more maternal licking and grooming as pups have reduced responses to stress and increased exploratory behavior in a novel environment [26, 27]. Given the importance of gentle affective touch in development, differences in somatosensory processing in ASD may be responsible for some aspects of autism-related phenotypes. Our knowledge of different populations of touch neurons and their projections in the brain provides direction on where we might begin this research.

In the hairy skin, several low-threshold mechanoreceptors (LTMRs) wrap around hair follicles to mediate the sense of innocuous touch. Of the Aβ, Aδ, and C subtypes of LTMRs, C-LTMRs are unmyelinated, found only in hairy skin, and are optimally tuned to stroking of skin at rates that are deemed pleasurable and at temperatures near those of human skin [28–32]. Further, firing frequency of C-LTMRs positively correlates with psychological rating of touch “pleasantness” [29, 33]. C-LTMRs are therefore implicated in affective social touch (i.e. touch between a parent and a child or between intimate partners) in humans. The brain regions implicated in processing C-LTMR-mediated touch (e.g. insular cortex) are also implicated in social perception and social cognition, processes in which individuals with ASD often display deficits [1, 29, 30, 34]. Further, there is a striking association between social abilities, touch preferences, and brain mechanisms for processing affective touch [35]. Individuals with more autism-related traits have reported an aversion to social touch, complementing a negative correlation between autism-related traits and brain responses to affective touch [35]. Furthermore, participants with more autistic traits exhibited less activation to C-LTMR-mediated gentle touch in the “social brain,” a set of brain regions that have evolved to support social cognition including the amygdala, orbitofrontal cortex, and temporal cortex [35, 36]. These results suggest that somatosensory deficits and sociability deficits are linked, but only via correlational evidence and without an analysis of how autism-related genes may play a role in these deficits. There are several genetic rodent models of ASD that exhibit aberrant tactile processing, allowing us to begin to explore how autism-related genes may contribute to the ASD phenotype by impacting the somatosensory system.

## Perception of Tactile Stimulation: From Skin to Brain

**Fig. 1:**
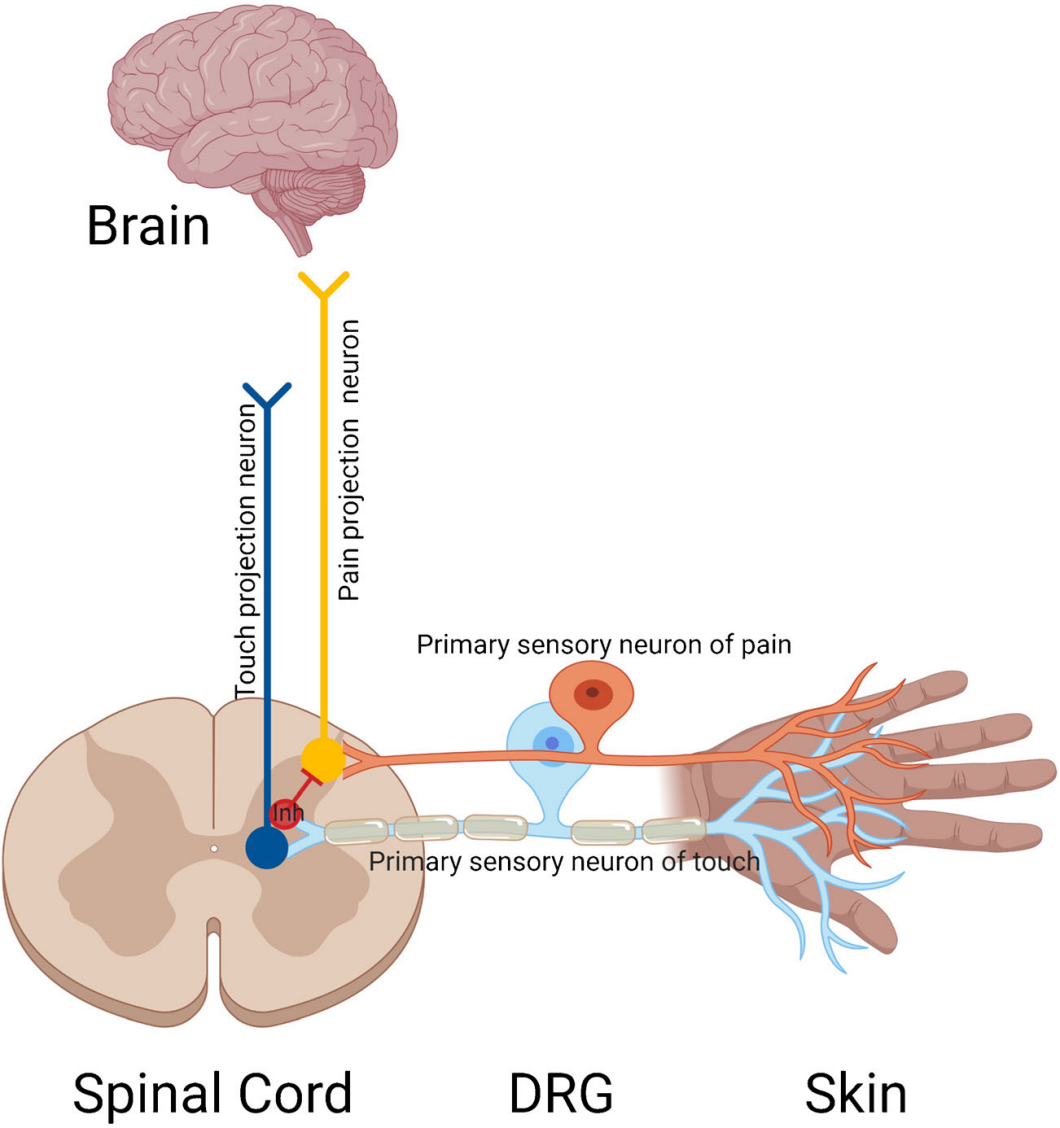
Pseudo-unipolar sensory neurons in the dorsal root ganglion project one sensory neurite to the skin and another to the dorsal horn of the spinal cord. Discrete subtypes of primary sensory neurons detect noxious and innocuous mechanical stimuli and ultimately activate distinct projection neurons which deliver somatosensory information to the brain. However, interneurons in the spinal cord add complexity to this circuit: touch neurons do have input to pain projection pathways, but local interneurons keep this under strong inhibition under healthy conditions

In order to adequately discuss the current research on the somatosensory system in autism models, we must first gain an appreciation for how our nervous systems encode somatosensory stimuli. The somatosensory system provides mammals with the capacity to explore their environment and form meaningful social bonds via tactile stimulation of the skin, while remaining safe from physical harm. In the peripheral nervous system (PNS), dorsal root ganglion (DRG) and trigeminal ganglion (TG) pseudo-unipolar primary afferent neurons project one sensory neurite to the skin and another to the dorsal horn of the spinal cord, thus connecting the periphery to the central nervous system (CNS) (Fig. 1). The multitude of DRG and TG neurons that respond to not only tactile stimulation but also thermal and itch stimuli has distinct nerve fiber diameters, conduction velocities, gene expression profiles, sensory ending morphologies, depth in the skin, rate of adaption to sustained stimulus application, and unique innervation patterns in both the skin and spinal cord. In this review, we will focus primarily on encoding of cutaneous stimuli in the periphery in the context of autistic phenotypes.

The perception of touch is sensed in the skin by four types of low-threshold mechanoreceptive sensory neurons (LTMRs) that terminate in elaborate end-organ structures composed of intertwined wrappings of epithelial or Schwann cells. As mentioned briefly above, LTMRs are subdivided by functional properties such as soma size and gene expression patterns, into classes known as Aδ-, Aβ-, and C-LTMRs. In general, large-diameter, heavily myelinated neurons (Aβ fibers) respond to touch, while smaller diameter, lightly, or unmyelinated neurons (Aδ or C fibers) respond to pain. However, Aδ-LTMRs and C-LTMRs are exceptions to this generalization, as they are subsets of intermediate- and small-diameter neurons that respond to touch rather than pain. Aδ- and Aβ-LTMRs encode discriminative touch, which is the ability to detect fine structural details of an object with our fingers, for example [37, 38]. A second type of touch, known as affective touch, occurs in a social context typically between friends, lovers, and family and is encoded by C-LTMR neurons [39–42]. In contrast to the perception of touch that predominantly utilizes special end organs, painful, potentially damaging stimuli are generally sensed by “nociceptors” which innervate the skin with free nerve terminal endings, devoid of attachment to any sensory end-organ [43]. A recent groundbreaking study however has updated our classification of nociceptive sensory cells in the skin by demonstrating in mice that a specialized Schwann cell associated with nociceptors forms a glio-neuro end-organ structure that is capable of transducing mechanical and thermal noxious stimulation [44].

The spinal cord serves as the relay station between the skin and brain, and is the first location where the body prioritizes how much touch versus pain information reaches the brain and our conscious awareness. Interneurons in the dorsal horn of the spinal cord control the crosstalk between “touch” and “pain” labelled lines from the periphery organized in different laminae of the spinal cord dorsal horn [45–52] (Fig. 1). Touch information from the skin typically enters in deeper spinal laminae within the dorsal horn, while nociceptive information enters in more superficial spinal laminae of the dorsal horn. Touch signals are sent to the brain directly from some Aβ-LTMRs to the gracile nucleus of the medulla [53], or indirectly from projection neurons that ascend ipsilaterally to the brainstem dorsal column nuclei [54]. Pain signals are sent to the brain from the spinal cord from long-range projection neurons that first cross over to the opposite side of the spinal cord followed by ascending directly to the brain in five major ascending pathways termed spinothalamic, spinoreticular, spinomesencephalic, cervicothalamic, and spinohypothalamic [55]. Extending from the thalamus, tactile information is encoded in the somatosensory cortex in the post central gyrus just three synapses away from receptors in the skin. In regard to pain, while the thalamocortical projections are important for the discriminatory components of the pain response, the negative valence state associated with pain is encoded in brain structures that receive thalamic input such as the basolateral amygdala and anterior cingulate cortex [56, 57].

The findings described above provide a basis for investigators to research how the peripheral nervous system and various ascending pathways may be altered in ASD. With the ability to target, observe, and manipulate specific populations of touch or pain neurons in mouse models, researchers have begun to study the PNS in the context of autism.

## Rodent Models Relevant to Syndromic ASD With a Somatosensory Phenotype

Animal models are highly useful in understanding the contribution of genetic mutations to tactile abnormalities. With rodent models, we can apply different noxious and innocuous stimuli in a controlled setting and objectively assess behavioral and physiological responses. Rodent models also allow us to control for any environmental influences that can contribute to an ASD phenotype. Further, neural mechanisms underlying sensory processing are well-conserved between humans and rodents, and several genetic rodent models relevant to idiopathic and syndromic forms of ASD display abnormal sensitivity to somatosensory stimuli (Table 1) [58].

**Table 1:** Selected studies of autism-related genes and how they contribute to a somatosensory phenotype

Study	Year	ASD-related gene	Tactile behavioral phenotype	Molecular/physiological mechanism	Source of phenotype
Han et al.	2016	SHANK3	Shank3 knockout → impaired heat hyperalgesia	SHANK3 regulates TRPV1 function and expression	Peripheral
He et al.	2017	FMR1	Fmr1 knockout → hypersensitivity to tactile stimuli	Layer 2/3 neurons in barrel cortex show reduced response to whisker stimulation	Central
McCoy et al.	2017	UBE3A	Whole genomic deletion of maternal Ube3a → increased sensitivity to noxious heat and mechanical stimuli	DRG-specific maternal deletion of Ube3a → normal sensitivity	Central
Bhattacherjee et al.	2017	MECP2	Mecp2 knockout → hypersensitivity to mechanical stimuli	Increased nonpeptidergic innervation of cutaneous targets in Mecp2 knockouts	Peripheral
Orefice et al.	2016	MECP2	DRG-specific deletion of Mecp2 → abnormalities in touch sensitivity and cognitive and social deficits	DRG-specific Mecp2 deletion → decreased GABRB3 in dorsal horn of spinal cord → decreased PSI of LTMR inputs to CNS	Peripheral

For example, mice with a deletion of the entire SH3 and multiple ankyrin repeat domains 3 (SHANK3) protein coding sequence display behavioral phenotypes that resemble the major features of SHANK3-related ASD, including pain sensitivity [59, 60]. SHANK3, like other members of the SHANK gene family, is a scaffold protein that plays a role in synapse formation and dendrite maturation [61]. It is expressed in both human and mouse DRG neurons, including LTMRs [62, 63]. The gene is highly implicated in ASD, with mutations occurring in ~ 2% of all patients [64]. Approximately 77% of individuals with Phelan-McDermid syndrome, a genetic condition with symptoms of ASD and frequently co-occurring with an ASD diagnosis, in which the entire SHANK3 gene is deleted, demonstrates decreased pain sensitivity, suggesting that the gene plays a role in pain processing [65]. Han et al. found that *Shank3* is broadly expressed in mouse DRG neurons and their terminals in the spinal cord. Strikingly, they also found that *Shank3* plays a role in pain transduction via regulating transient receptor potential ion channel subtype V1 (TRPV1), a capsaicin receptor that regulates heat transduction and is expressed in C-fiber nociceptive neurons [60]. The currents recorded from TRPV1 channels in DRG neurons were reduced in the *Shank3* mutant mice, complementing the impaired heat hyperalgesia observed behaviorally in these knockout mice. They also found that haploinsufficiency of SHANK3 causes defects in TRPV1 function in both mouse and human DRG neurons. These results indicate that SHANK3 mutations can alter the function of peripheral neurons, suggesting a possible peripheral mechanism for somatosensory deficits in ASD.

Fragile X syndrome (FXS) is another common syndromic form of ASD (~ 2% of ASD cases), with symptoms including intellectual disability, repetitive behaviors, social deficits, increased anxiety, and abnormal sensory processing [66]. FXS is caused by a mutation in the fragile X mental retardation 1 (FMR1) gene, which codes for fragile X mental retardation protein (FMRP). FMRP is implicated in synaptic protein synthesis and synaptic plasticity [67, 68]. *Fmr1* knockout mice display several behavioral deficits similar to human FXS symptoms including hypersensitivity to tactile stimuli [69, 70]. While investigating whether these tactile deficits were due to abnormalities in the somatosensory cortex, He et al. found that the proportion of layer 2/3 neurons in the barrel cortex (a region of the somatosensory cortex) that respond in a time locked manner to whisker stimulation is 45% lower in *Fmr1* KO mice, and that L2/3 neuronal activity in the KO mice shows a lack of adaptation to repetitive whisker stimulation [69]. These results suggest that it is a lack of neuronal adaption to tactile stimuli in the somatosensory cortex that contributes to the sensory overactivity in FXS, and perhaps in other ASDs. More work is needed to determine whether the loss of adaptation to tactile stimuli originates in the periphery and projects to the somatosensory cortex where the differences were observed, or whether the adaptation deficit occurs solely in the brain.

Another syndromic form of ASD is Angelman syndrome (AS), a severe neurodevelopmental disorder caused by a mutation or deletion of the maternal UBE3A allele [71]. The ubiquitin-protein ligase E3A (UBE3A) gene codes for the UBE3A enzyme, which tags proteins with ubiquitin to target them for degradation. UBE3A is expressed on both alleles in most tissues, but the full-length protein is expressed primarily from the maternal allele in the CNS [72–74]. UBE3A displays differential allelic expression because paternal UBE3A is repressed in the CNS by UBE3A-ATS, a long noncoding transcript [75–78]. Little research has been done investigating the expression pattern of UBE3A in the PNS, but parent reports and questionnaires indicate that sensory processing deficits are also common in AS, including slow responses to pain [79, 80]. McCoy et al. recently found that large-diameter touch neurons in the DRG expressed maternal *Ube3a* and paternal *Ube3a-ATS*, while most small-diameter pain neurons expressed Ube3a on both alleles with little levels of Ube3a-ATS [81]. In short, touch neurons express Ube3a protein from the maternal allele, similar to the CNS, while pain neurons express the protein from both alleles. Additionally, behavioral responses to noxious stimuli were enhanced in AS model mice, in which there is a deletion of maternal Ube3a. Interestingly, a conditional deletion of maternal Ube3a in only the DRG did not affect responses to painful mechanical stimuli. These data indicate that the enhanced nociceptive responses in AS mice are due to loss of maternal Ube3a in the central and not the peripheral nervous system, suggesting a central mechanism for tactile deficits in AS.

While a loss-of-function mutation in UBE3A underlies Angelman’s syndrome, gain-of-function mutations of UBE3A are also associated with autistic features. A central mechanism implicating the autism-liked gene, cerebellin1 (CBLN1), was recently proposed to underlie these UBE3A-mediated social deficits [82, 83]. Krishnan et al. demonstrate that the increase in UBE3A induces a downregulation of CBLN1, and further, that behavioral changes following loss of CBLN1 specifically in ventral tegmental area (VTA) neurons mimic the UBE3A-mediated social deficits. CBLN1 is a synaptic organization protein that, while primarily studied for its role in motor function in the cerebellum, is also implicated in learning deficits through its function in the hippocampus [84–86]. While these findings suggest that a central mechanism explains the CBLN-mediated behavioral features of autism, because peripheral changes in these models are unexplored, a peripheral mechanism cannot be excluded. High-throughput single-cell RNA sequencing studies reveal that CBLN1 is highly co-expressed in one small subset of DRG neurons that also express mas-related g-protein coupled receptor B4 (MrgprB4) [87, 88]. Interestingly, the MrgprB4-expressing neurons are thought to represent a population of C-LTMRs and therefore are speculated to play a role in social touch [89]. Thus, while the focus for CBLN1 has been central, it remains an interesting possibility that autism-linked CBLN1 deficits also impair sociability by altering sensation of socially relevant tactile stimuli by its function in a subset of C-LTMRs.

Rett syndrome (RTT), another severe autism-associated developmental disorder, is caused by mutation of the X-linked gene, methyl-CpG binding protein 2 (MECP2) [90]. The MECP2 protein regulates protein synthesis by acting as a transcriptional repressor or activator [91]. Interestingly, there is evidence in zebrafish that well-known axon guidance cues Semaphorin 5b (Sema5b) and Roundabout Guidance Receptor 2 (Robo2) are positively regulated by Mecp2 and are needed for innervation of sensory neurons [92]. It is therefore no surprise that individuals with RTT have somatosensory abnormalities, with reports of both insensitivity and elevated pain thresholds [93–95]. While investigating how the MECP2 gene might affect the PNS in mammals, Bhattacherjee et al. found that a rat model of RTT displayed increased pressure and cold sensitivity, hyposensitivity to heat, and interestingly, increased DRG axon outgrowth [96]. Further, MeCP2 knockdown specifically in DRG neurons increases DRG axon outgrowth and causes mechanical hypersensitivity in wild-type rats [96]. These findings suggest that MECP2 plays a major role in regulating peripheral innervation, indicating a peripheral mechanism for the tactile abnormalities seen in RTT.

**Fig. 2:**
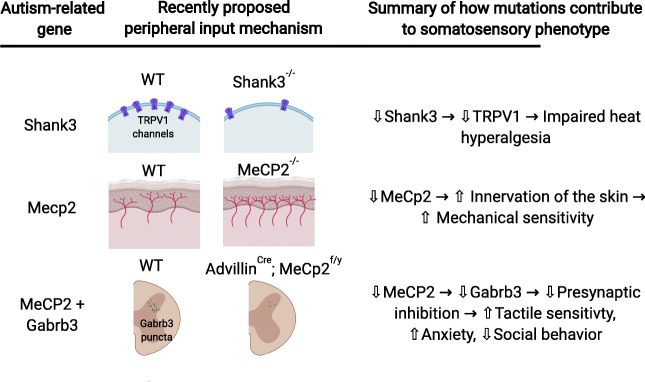
Examples of how mutations in autism-related genes may contribute to tactile abnormalities. Based on results from Han et al. 2016 [60], Bhattacherjee et al. 2017 [96], and Orefice et al. 2016 [98]

Rodent models of syndromic ASDs have been a valuable tool in understanding the social, cognitive, and sensory aspects of these neurodevelopmental disorders. While major strides have been made in recent years, we still have a lot to learn about the role that ASD-related genes play in tactile sensitivity, and how tactile sensitivity may contribute to the behavioral symptoms of ASD. The results from the aforementioned MeCP2 and Shank3 studies, along with others, suggest that peripheral mechanisms are responsible for the tactile differences in these disorders (Fig. 2) [60, 96]. Recently, labs have begun to focus on how peripheral mechanisms may contribute to the social and repetitive core symptoms of autism.

## The Role of the PNS in Contributing to Behavioral Symptoms of ASD

The majority of ASD research has focused on brain-specific mechanisms and circuits, with little attention to potential contributions of the PNS to ASD phenotypes. A recent review by Cascio et al. describes in depth how social touch in development is highly important for the formation of secure attachment, cognitive and linguistic development, and social reward [97]. Normal somatosensory functioning during development may therefore be essential for later expression of normal social behaviors. Recent groundbreaking studies by Orefice et al. sought to investigate how mutations in autism-related genes may contribute to tactile sensitivity abnormalities, and how these abnormalities may contribute to an ASD diagnosis [98, 99].

Similar to results from other researchers, Orefice et al. found that mice with mutations in *Mecp2*, *Shank3*, *Fmr1*, and gamma-aminobutyric acid receptor subunit beta-3 (*Gabrb3*) all display altered tactile discrimination and hypersensitivity to gentle touch [69, 96, 98, 99]. These mutant models were selected based on their genetic and behavioral similarities to humans with autism. Strikingly, tactile deficits caused by Cre-lox mediated developmental deletion of *Mecp2*, *Gabrb3*, or *Shank3* specifically in DRG neurons led to behavioral deficits resembling some of the core phenotypes of ASD (anxiety-like behaviors, reduced sociability, and reduced preferences for social novelty) [98, 99]. Deletion of *Mecp2* and *Gabrb3*, a GABA_A_ inhibitory receptor subunit, in adult DRG neurons led to similar changes in tactile sensitivity but resulted in more modest social deficits [98]. Deletion of Shank3 at P28, as opposed to earlier in development, also led to tactile abnormalities, and less severe social impairments [99]. These findings suggest that *developmental* somatosensory dysfunction caused by mutations in autism-related genes can cause ASD core symptoms. A decrease in the number of parvalbumin-positive (PV+) neurons in the basolateral amygdala, a brain region involved in anxiety and social behaviors, in mice with DRG-specific Shank3 and Mecp2 mutations suggests a potential mechanism for why periphery-specific mutations of ASD-related genes may result in anxiety behaviors and social deficits [99].

Because deletion of *Mecp2* in the DRG led to a decrease in *Gabrb3* in the dorsal horn of the spinal cord, the authors proposed a mechanism for how Mecp2 mutations lead to somatosensory abnormalities: reduced presynaptic GABA-mediated inhibition of LTMR inputs to the CNS [98]. In a follow-up study, they also found that large-diameter neurons cultured from Shank3 mutants displayed decreased current mediated by hyperpolarization-activated cyclic nucleotide-gated (HCN) channels and increased excitability compared with neurons harvested from control mice [99]. Together, these findings suggest that there are at least two mechanisms that underlie tactile hypersensitivity in ASD models: GABA- and HCN-mediated. Therefore, in attempting to treat tactile or other abnormalities in ASD by targeting the peripheral nervous system, it is unlikely that there will be a one-size-fits-all method.

Orefice et al. have already begun investigating the potential therapeutic benefit of targeting peripheral somatosensory neurons in ASD models [99]. Restoration of Shank3 or Mecp2 in cells below the neck or exclusively in DRG neurons was able to rescue some sensitivity deficits, anxiety-like behavior, and social deficits. Postnatal viral restoration of Gabrb3 in PNS neurons of mice with PNS-specific Mecp2 mutations improved tactile abnormalities, anxiety-like behaviors, social impairments, and PV+ neuron abnormalities in the BLA. Perhaps most strikingly, peripherally restricted pharmacological treatment with a GABA_A_ receptor agonist reduced hairy skin hypersensitivity in Shank3, Fmr1, and Cntnap2 mutant mice and a mouse model of maternal immune activation-induced (MIA) ASD. It also improved anxiety-like behaviors, social impairments, and PV+ neuron abnormalities in the BLA in Shank3 and Mecp2 mutant mice without causing sedation. These results suggest a potential novel therapeutic strategy for treating certain ASD-related behaviors.

## Conclusion

Historically, research on the etiology of ASD has focused heavily on the brain, but peripheral mechanisms of ASD are beginning to be uncovered, with growing evidence that somatosensory neurons are dysfunctional and contribute to ASD-related behaviors in a range of ASD models including *Mecp2*, *Gabrb3*, *Shank3*, *Cntnap2*, and *Fmr1* mutants, and MIA models [96, 98, 99]. With evidence that DRG-specific loss of ASD-related genes can lead to changes in brain circuits, particularly the number of PV+ interneurons in the somatosensory cortex and BLA, in addition to ASD-related behaviors, early therapeutic targeting of the PNS could be the future of autism treatment research.

It is important to note that loss of these ASD-related genes in the PNS has not yet been shown to cause any repetitive behaviors or memory deficits, which are common ASD-related symptoms. It is likely that a combination of mutations in the CNS and the PNS contributes to the full spectrum of behavioral deficits. However, evidence that we can treat multiple symptoms of ASD by targeting the PNS provides a basis for the future of autism therapeutic research. By targeting the PNS, we can circumvent side effects associated with directly manipulating brain circuits, such as sedation, as many of the current FDA-approved drugs that cross the blood-brain barrier do [99–101].

As sensory abnormalities in ASD are not restricted to somatosensation, it is possible that, similar to the findings by Orefice et al. [98, 99], abnormalities in other senses could lead to abnormal social behaviors. For example, auditory hyper- or hyposensitivity could result in deficits in language learning and production. Sensory hypersensitivity may also contribute to other ASD symptoms such as anxiety, hyperarousal and sleep deficits, attention deficits, stereotyped behaviors, and learning disabilities. For example, people have reported fluorescent lights increasing repetitive behaviors or causing tantrums [102]. Further studies are needed to examine how sensory abnormalities may contribute to the ASD-related behaviors, and could ultimately lead to the development of novel approaches to early intervention and treatment.
