# Correction to: Mechanisms of Tactile Sensory Phenotypes in Autism: Current Understanding and Future Directions for Research

**DOI:** 10.1007/s11920-022-01345-0

**Published:** 2022-06-18

**Authors:** Melanie D. Schaffler, Leah J. Elias, Ishmail Abdus-Saboor

**Affiliations:** Department of Biology, University of Pennsylvania, Philadelphia, PA 19104 USA

## Correction to: Current Psychiatry Reports (2019) 21: 134 https://doi.org/10.1007/s11920-019-1122-0

In the version of this article originally published, the name of the author Leah J. Middleton should be updated to Leah J. Elias due to change of marital status.

